# Relationship dynamics and anticipated stigma: Key considerations for PrEP use among Tanzanian adolescent girls and young women and male partners

**DOI:** 10.1371/journal.pone.0246717

**Published:** 2021-02-17

**Authors:** Nrupa Jani, Sanyukta Mathur, Catherine Kahabuka, Neema Makyao, Nanlesta Pilgrim

**Affiliations:** 1 Population Council, Washington, DC, United States of America; 2 CSK Research Solutions, Dar es Salaam, Tanzania; 3 National AIDS Control Programme, Dar es Salaam, Tanzania; 4 Washington, DC, United States of America; Simon Fraser University, CANADA

## Abstract

**Objectives:**

We examined key gender, interpersonal and community dynamics influencing PrEP acceptability among adolescent girls and young women (AGYW) and their male partners.

**Methods:**

We administered 12 in-depth interviews (IDI) to partnered, or married AGYW aged 15–24 years living without HIV, and 16 IDIs to male partners living without HIV aged 18 or older, partnered or married to an AGYW in Tanzania. Card sorting, a participatory qualitative method for facilitating systematic discussion, was used to identify attitudes, values, and desires that would influence PrEP acceptability.

**Results:**

Relationship distrust, partner communication about HIV risk, and need to control HIV risk were highly influential considerations for PrEP use. AGYW and male partners both wanted to discuss PrEP use amidst relationship distrust, while most male partners encouraged AGYW PrEP use for shared protective benefit. Anticipated stigma of being perceived as a person living with HIV, as a result of PrEP use, was a deterrent for both AGYW and male partners while AGYW also feared additional stigma of being considered sexually promiscuous.

**Conclusions:**

Couples counseling for PrEP uptake and adherence might be a well-placed strategy for couples who are living without HIV to educate one another about the relationship benefits of using PrEP, thereby increasing its acceptance and adherence, addressing unequal power dynamics, and reducing associated relationship distrust. Community awareness and education about PrEP can help curb persistent PrEP stigma, including intersectional stigma.

## Introduction

Adolescent girls and young women (AGYW, women aged 15–24 years) remain highly vulnerable to HIV across sub-Saharan Africa (SSA) [1]. AGYW bear a heavy burden of HIV compared to their male peers and HIV-related mortality remains the leading cause of death among AGYW in the region [2]. In Tanzania, specifically, new infections among AGYW aged 15–24 years are occurring at a rate twice as high as those among men, in 2018 at 16,000 compared to 7,600 respectively [3]. HIV prevalence is also three times higher in young women (4.4 percent) compared to young men (1.1. percent) [2, 4, 5]. Oral pre-exposure prophylaxis (PrEP)–daily HIV medicine taken by people who are living without HIV to avoid getting HIV–can help stem the HIV epidemic among AGYW with effective access and use. However, since its introduction in 2010, PrEP uptake has been slow. To date, only 71 countries have approved use of PrEP and globally there are approximately 465,000–475,000 users of PrEP [6], falling far short of the UNAIDS global target of reaching 3 million people with PrEP by 2020 [7]. Likewise, access to and uptake of PrEP remain low among AGYW across SSA.

PrEP is one of few biomedical HIV prevention methods that AGYW can use and control by themselves, and if used effectively could substantially alter HIV acquisition among AGYW. Yet, a recent systematic analysis that examined 18 global, prospective randomized control trials and PrEP implementation studies found highly variable adherence to PrEP among women, ranging from 29–76% [8]. A study in Kenya showed only 22% of young women offered PrEP started using it, even though 39% did not know their partners’ HIV status [9]. Another PrEP project in Kenya found the drop-out rate among young women was nearly 75% after just one month of enrolment [10]. A range of individual, interpersonal, structural, and socio-cultural factors can influence PrEP uptake and use [11]. Previous studies have found that adolescents can be reluctant to seek sexual and reproductive health (SRH) services due to experienced or anticipated stigma and discrimination from healthcare providers, feeling socially marginalized, or a lack of social support [12, 13]. While recent studies have shown the important role that community-based organizations [14], networks [15], and health service providers [13] play in increasing awareness and support around PrEP use and uptake [16], few have assessed gender and power dynamics, specifically within interpersonal, partner, and community relationships that may affect PrEP use for AGYW. Current efforts to implement PrEP are hampered by low uptake and adherence [17]. Of note, there were only 3,200–3,700 reported PrEP users in Tanzania as of October 2019 [18].

AGYW vulnerability to HIV is in large part due to relationship and power dynamics, including gender and social norms that dictate associated behaviors [19], having older sexual partners, difficulty negotiating safer sex practices [4], intimate partner violence [2, 20] and engagement in transactional sex [21]. At the same time, women and AGYW’s retention in prevention of mother to child transmission programming and use of condoms has been shown to increase with male partner involvement [22] while their access to contraceptives has proven to be much more challenging without the support or accompaniment of a male partner [23]. Given the important role and influence male partners may have in AGYW’s PrEP decision making, it is vital to understand their perspectives and attitudes towards PrEP to inform PrEP uptake strategies. Yet, most studies on PrEP use and acceptability among men have been conducted among men who have sex with men (MSM), gay, transgender, and bisexual men [24, 25]. Less is known about the views of heterosexual men in SSA with regards to taking PrEP as well as views on their partner’s PrEP use. More information is needed to understand how gender and power dynamics, including their manifestation within interpersonal and partner relationship dynamics may affect PrEP use among AGYW and their male partners. Importantly absent from the current literature on PrEP use is an understanding of how the asynchronous targeting and rollout of PrEP among the high-risk populations mentioned may affect its uptake among other vulnerable populations, such as within the context of AGYW relationships.

In this study, we assess key relationship and contextual factors influencing the uptake and adherence to PrEP as outlined in the framework for PrEP introduction for AGYW [11] to explore critical considerations for PrEP acceptance and use among AGYW and male partners of AGYW in rural and urban Tanzania. At the time of this study Tanzania was considering introducing PrEP. It has been established that formative evaluations that are designed to address anticipated facilitators and barriers can help policy makers and health care providers increase the possibility of successful implementation and uptake of interventions [26–28]. We conducted this acceptability study to understand AGYW and male partners’ perspectives on HIV risk to help inform the implementation of PrEP across the country. Specifically, we examine key interpersonal, partner, and community dynamics outlined in the PrEP Introduction Strategies for AGYW framework by Mathur et al. [11]. The framework outlines the dynamic interplay of factors that influence PrEP introduction for AGYW: 1) factors influencing the feasibility of PrEP introduction and providing quality services (i.e., scientific context, political landscape, health system infrastructure, and provider dynamics) and 2) key factors that inform choice, demand and effective use of PrEP among AGYW (i.e., AGYW characteristics, partner dynamics, family dynamics, community context). This paper examines those factors affecting AGYW’s ability to make informed choices in order to increase demand for and effective use of PrEP. Primarily we focus on the following aspects of partner dynamics and AGYW characteristics: HIV risk perception, social support, perception of stigma, gender norms, parental engagement, acceptability, and education and communication messaging. We compare and contrast the responses of AGYW and male partners to understand similarities and differences in their perspectives about PrEP.

## Methods

### Study design and sampling

This formative assessment took place in an urban district in Dar es Salaam and a rural district in Mbeya, Tanzania. These sites were purposively selected as they represented urban and rural locations with high HIV prevalence and where DREAMS (Determined, Resilient, Empowered, AIDS-free, Mentored and Safe), a large-scale multi-sectoral HIV-prevention program, was on-going. Data used for this study comes from a larger mixed-methods study exploring PrEP feasibility and acceptability in Tanzania, described elsewhere [13]. This analysis focuses on the subset of married or partnered AGYW. Twelve in-depth interviews (IDIs) were conducted with sexually active, married or partnered AGYW aged 15–24 years living without HIV, and 16 IDIs with male partners aged 18 or older, living without HIV, married to or living with an AGYW. Sample sizes were determined based on literature suggesting sufficient data saturation in qualitative studies [29]. Partnered AGYW were recruited through referrals from local civil society organizations implementing the DREAMS program or from health service provider referrals. Male partners were recruited through civil society organizations, health service provider referrals, and from partnered AGYW who did and did not participate in the study. An equal number of AGYW and male partners were selected from each location. IDIs were conducted from March to June 2017 shortly before PrEP was rolled out in country [30]. We focus on the perspectives of AGYW and male partners who report having AGYW partners.

### Procedures

To ensure that participants were adequately informed about PrEP, trained research assistants provided detailed information on oral PrEP to all participants using a visual, standardized PrEP script along with PrEP-like pills for demonstration. Research assistants conducted the IDIs in Kiswahili, which lasted approximately 45–60 minutes using a standardized guide.

Both AGYW and male partners were asked about perceptions of their and their partner’s risk for HIV, potential PrEP use by themselves and their partners, and concerns about using PrEP. Interview guide questions were informed by key domains and relationships between the key populations for informed choice, increased demand and effective use of PrEP based on the conceptual framework by Pilgrim et al. [31]. As PrEP was not available in Tanzania at the time of this study, we used card sorting, a participatory qualitative method shown to facilitate systematic discussion [32, 33], during each respondent interview to help concretize the issues being discussed and to generate insights around anticipated barriers and facilitators to PrEP use, reflecting on the relative importance of the concept to them. The research team developed the key constructs on the cards and the associated illustrations based on current literature around key factors influencing PrEP use and adherence [31, 34] to provide contextual validity. The cards were pilot tested during interviews among the Tanzanian based research team and then revised accordingly before they were finalized and implemented. The first section of the IDI posed open ended questions on HIV risk perception & avoidance strategies. In section two participants were read a short script on the purpose, use, and side effects related to PrEP, and then asked to sort the constructs on each of the cards as per the top three most, somewhat, and least important to determining their potential use of PrEP (see Table 1). Issues that participants highlighted were prioritized within the card sort activity and framed the remainder of the interview. Male partners and AGYW were shown nine cards noting key social factors influencing PrEP use [31] and were asked to select the top three cards that would most influence their PrEP use, then select the next three cards that would somewhat influence, and finally to select the remaining three cards that would least influence their use of PrEP. The cards included factors such as: wife/partner feelings about taking PrEP; family/parent’s feelings about taking PrEP; people will think I have HIV; and partner/wife’s HIV status. After ranking and prioritizing the cards, participants explained in detail their reasoning for selecting each card and its associated level of influence on their willingness and ability to take PrEP. Table 1 presents illustrative interview questions asked of both AGYW and male partners.

**Table 1:** Sample of in-depth interview guide questions asked to AGYW and male partners.

IDI guide section	Illustrative Questions
**SECTION 1: RISK PERCEPTION & AVOIDANCE STRATEGIES**	• Some people think that it is very likely that they will get HIV and some people think that they will never get HIV. What about you? How likely do you think it is that you will get HIV? Why do you say so? • Do you think that your sexual partner(s) might be at risk for acquiring HIV? Why or why not? • In general, what do you do to prevent yourself from becoming infected with HIV? What methods do you use?
**SECTION 2: Reactions to PrEP**	• Would you be interested in taking PrEP? Why or why not? • How would it make you feel to start taking PrEP? (please describe)
**Questions related to specific cards on key considerations for PrEP use**	
**‘Wife/partner feelings’**	• Why did you select ‘Wife/partner feelings’ as ____ important? • Would you tell your wife/partner that you decided to take PrEP? Why or why not? • If your partner did not want you to take PrEP, how would that influence your decision to take PrEP? • If your partner did not want you to take PrEP, what might change his/her mind?
**‘People will think I have HIV’**	• Why did you select ‘People will think I have HIV’ as most important? • How would your community’s opinion about PrEP influence your opinions and decision to take PrEP? • Who in your community would have the greatest influence on your decision to take PrEP? Why? • Would you discuss PrEP with this individual? Why or why not?
**‘Partner/wife HIV status’**	• Why did you select ‘Partner/Wife’s HIV status’ as most important? • Based on what you know about your partner (s) HIV status, does that make you more/less willing to take PrEP? • Would you be willing to ask your partner to get tested for HIV? Why or why not?

### Analytical approach

All IDIs were audiotaped, transcribed from Swahili to English into Microsoft Word, and imported into ATLAS.ti (Version 8.0) for analysis. Trained research staff reviewed all transcriptions against their corresponding Swahili audios for content and translation accuracy. Lead study investigators drafted the initial codebook using inductive coding. Three initial transcripts were each independently coded by each of the five research team analysts and then repeated once more to ensure inter-coder agreement. Upon simultaneous review of the coded transcripts, codes were modified accordingly. The study team reviewed and discussed the coded data and emerging themes. After no additional themes emerged, the team finalized the codebook and applied the codes to the remaining transcripts. The analysis team met regularly to discuss coding modifications and application of codes across and within different cadre of participant transcripts, and clarify areas of disagreement to establish convergent validity [35]. Thematic content analysis was used to identify key factors influencing willingness to take PrEP. Codebook modification and thematic content analysis were both performed iteratively as codes and themes were refined based on additional transcript data. Saturation of themes was determined after comparing groups of codes and identifying emerging themes in a subset of male partner and AGYW transcripts and reviewing remaining transcripts until no additional themes emerged. For the card sort, a ranking system was applied to aggregate most, somewhat, and least influential factors across participants, wherein the “Least important” category received 1 point; “Somewhat important” category received 2 points, and “Most important” category received 3 points. Cards with the highest scores were selected as main themes for further analysis, as presented below with the accompanying discussion.

### Ethical approval

The study protocol was reviewed and approved by the Population Council Institutional Review Board (New York, USA), the National Institute of Medical Research, Medical Research Coordinating Committee (Dar Es Salaam, Tanzania), and the Mbeya Regional Medical Research Ethics Review Committee, (Mbeya, Tanzania). Participant privacy was ensured by assigning ID numbers to each unique interview and transcript, with identifying information recorded in a separate form only accessible to the research team. All participants were instructed not to use real names during the interview when referring to themselves or others. Audio recordings of interviews were stored in password protected computers, under lock and key, accessible only to authorized research staff at the research partner study office. The study team obtained written informed consent from all participants included in the study. All participants were reimbursed 11,000 Tanzanian shillings [$4.75USD] for their time.

## Results

Sociodemographic characteristics of participants are presented in Table 2. All respondents were married or in a committed or live-in relationship.

**Table 2:** Respondents’ socio-demographic characteristics.

	AGYW	Male partners
**Number**	12	16
**Age range**	19–24 years	19–42 years
**Median age**	21 years	26 years
**Highest education level attained**	7 Primary school	6 Primary school
	5 Secondary school	8 Secondary school
		2 Post Secondary

All respondents expressed a sophisticated awareness of their own risk of HIV as well as that of their sexual partner. As a result, all desired access to and noted that they would use PrEP. Three key themes were identified with regards to AGYW and male partner’s considerations for PrEP use: relationship dynamics, anticipated stigma, and associated implications for PrEP programming.

### Relationship dynamics: Communication, faithfulness, and controlling HIV risk

#### Discussion and disclosure of PrEP use

Several elements related to partner dynamics emerged as the most influential considerations for PrEP use among AGYW and male partners. AGYW saw PrEP as an HIV prevention option they could take independently of their partner and not have to rely on his actions, as in the case of condom use.

*Using PrEP is easier because it is just about myself. I can decide to use PrEP unlike condoms where you may want to use but your partner doesn’t*.

–AGYW, age 20, Mbeya

Yet, both male partners and AGYW noted the prime importance of their partners’ perspectives in their potential use of PrEP. All respondents wanted to discuss and disclose PrEP use with their partner. Speaking openly with their partners about an array of HIV prevention measures, including condom use and PrEP, would help convince their partners about the shared benefits of PrEP. When asked what her partner’s reaction would be to her decision to take PrEP, one AGYW replied:

“…if you give him clear instructions, he would join you in using PrEP.”

-AGYW, age 20, Mbeya

A few women confirmed that it would be difficult to use PrEP covertly and doing so would likely result in relationship discord. One young woman alluded that her relationship would be negatively impacted by her non-disclosure of PrEP use:

*Our relationship will be affected if he is not informed*, *but if I briefly explain it to him*, *he will understand I am using PrEP so as to protect myself from HIV infections… if he understands me properly*, *he can decide to accompany me and begin to take PrEP as well*. *But if I don’t tell him and he finds them on his own*, *that is where the problem steps in*.

–AGYW, age 22, Dar es Salaam

Reported potential relationship problems as possible outcomes of covert PrEP use ranged from relationship dissolution, to loss of financial support, to emotional or physical violence. A few AGYW noted their male partners might shout at or beat them if they took PrEP without notifying them beforehand or without their explicit knowledge and permission. Several male partners mentioned that they would divorce their partners if they found out she was taking PrEP without consultation. When a 30-year-old male partner from Mbeya was asked what his reaction would be to his partner taking PrEP without his knowledge he replied, *“For that only I could be mad at her…for this I will send her home…I could even beat her…it could affect us very much and I would even divorce her*.*”*

Men too wished to be informed about PrEP and be involved in the decision-making process to use it. As one male partner noted:

*First is…to involve me in what she wishes to do, which is to use these medications…the most important thing is for me to be involved in the whole process*.

–Male partner, age 42, Dar Es Salaam

Male partners wanted to have access to PrEP, which they felt would help them inform their partner and encourage use. When one male partner was asked if he would disclose his PrEP use to his partner he replied:

*Yes, I can tell her, and she must understand. I must teach her about it so that she may also understand so that she may allow me to take it*.

–Male partner, age 26, Mbeya

Similarly, male partners noted that they would also support their partner in taking PrEP for a variety of reasons, including sharing the benefit of being protected from HIV. When one respondent was asked if he would like for his partner to take PrEP, he replied:

*Yes*, *I would like*. . . . *Simply because I have understood about it*, *and*, *it is the best way to protect ourselves from HIV and keep us safe*.        –Male partner, age 28, Mbeya

#### Partner trust

While partner’s perspectives about PrEP use and desire for open communication ranked highly for AGYW and male partners, they also described high levels of distrust within their relationships. AGYW and male partners were both unsure of their partner’s sexual networks, stemming from perceived or experienced infidelities within the relationship. Therefore, both believed they were vulnerable to HIV and wanted to use PrEP.

*… my husband moves to different places and I can’t know if he has other partners*.

–AGYW, age 21, Dar es Salaam

Men acknowledged engaging in sexual relations with women outside of their primary partnership. They also reported that they either did not use condoms or used them inconsistently with their occasional or regular partners. With their regular partner, male partners noted condom use would signal a breach of trust. Thus, PrEP could play a vital role in protecting themselves and their partners from HIV. When one respondent was describing discussions with his partner about protecting themselves, his response was:

*…because I am taking PrEP*, *even if I cheat*, *I will not get HIV…because I am a young adult*, *I don’t have only one partner*, *so if she disagrees with me on taking PrEP*, *I might have another sexual partner and if she is HIV positive*, *I may get HIV; and hence she may also get HIV*.–Male Partner, age 24, Mbeya

Unlike male respondents, the majority of AGYW stated they were faithful in their relationships and ascribed their HIV risk to their partners’ behaviors. AGYW specifically spoke about their inability to negotiate sexual activity with their husbands or primary partners, as they were unsure about their partner’s faithfulness.

Things that will put me at risk for HIV are … my own partner because I will not be sure of what he is doing out there, he might be hanging out with other women. He is my husband he will need to have sex with me, I cannot resist him…

–AGYW, age 19, Mbeya

This underlying distrust in relationships furthered desires for partner support and candid communication about HIV prevention measures. Participants did not want to exacerbate infidelity concerns in their relationship which could potentially lead to negative consequences, but instead preferred having open dialogue about shared concerns.

*Yes, we have ever discussed about protecting ourselves from HIV. I told her that though we trust one another, there is time me as a man I am tempted by other girls, so she is advising me to be faithful and if I fail to obey that I must make sure that I am protecting myself. How? I must use protective measures to protect myself against HIV*.

–Male partner, age 30, Mbeya

#### Anticipated stigma: Family, peers & community perspectives

Both AGYW and male partners highly ranked peer and family perspectives and the potential for anticipated stigma as key considerations for PrEP use during the card sort. Since PrEP is an antiretroviral drug, both groups feared that prevailing stigma toward people living with HIV (PLHIV) would apply to them if they were to take PrEP.

#### Community influence on PrEP use

Anticipated stigma of being labeled as a person living with HIV was frequently mentioned by male partners. Male partners were concerned about their peers’ perceptions of their HIV status, noting that friends would make fun of them for using PrEP. When a 24-year-old driver from rural Mbeya was asked how his community would react to young men his age taking PrEP, he noted, “*They will start laughing at me…because they will think that I am infected which is not true*.*”* Male partners also commented on the mental health effects of being perceived as living with HIV, which included feeling a loss of manpower, anxiety, and alienation from their family and peers. As one respondent noted:

*…when they [people] see you taking PrEP, they will think that you are taking ARVs. So, this is going to affect me psychologically… As you know, our parents and the community at large are not well educated, so by taking PrEP they may think that I have HIV…[and]… then you are going to be discriminated*.

–Male partner, age 26, Mbeya

AGYW were also concerned about anticipated HIV stigma from their peers and communities. Women noted that their friends perceive and treat PLHIV poorly and try to distance themselves for fear of transmission. One young woman explained:

*Some when they see a HIV infected person*, *they think he/she was a prostitute or had bad sexual behaviors… Some will keep their relationship with me*, *others will stigmatize me thinking I am HIV infected*.–AGYW, age 21, Mbeya

#### Family influence on PrEP use

Women acknowledged that anticipated HIV-related stigma would pose a barrier to their potential PrEP use. Opinions of family emerged as especially important to AGYW with regards to potential PrEP use. AGYW expressed complicated feelings about anticipated HIV stigma from their parents. While AGYW wanted to be honest with their parents about their potential use of PrEP, they also did not want to be judged for protecting themselves from HIV. While some women mentioned that their family members are understanding and accepting of people living with HIV, others noted that if they were to acquire HIV, their parents would assume that it was due to promiscuous sexual behaviors, which in turn had implications on their potential use of HIV prevention medicines.

*They [my family members] consider them [people living with HIV] as prostitutes*, *and that is why they acquired HIV*.–AGYW, age 22, Dar es Salaam

*…parents might say ‘Who told you to use this medicine*?*’ They might throw them away while you are intending to protect yourself…Personally*, *I cannot go and tell my father that I am going for PrEP services*. *He will stop me and ask*, *‘who said that you are sick*?*’–*AGYW, age 20, Mbeya

Of note, although AGYW were anticipating stigma from parents, they also desired parental support for their use of PrEP.

*I will tell my mother [that I am using PrEP] because she is my first parent… She might want to know what PrEP is and what it does. So, I will sit down to tell her and explain well. She will understand me and allow me to use it*.

–AGYW, age 21, Dar es Salaam

#### Considerations for PrEP education programming: Health provider preference & community sensitization

To build partner engagement and greater communication around PrEP, AGYW and male partners cited the need for early and comprehensive education about the uses, benefits, and side effects of PrEP. They desired this information to enhance their mutual understanding. Male partners highlighted the importance of receiving accurate information about PrEP from health service providers, noting they could suggest strategies to actively support their partner’s adherence to PrEP, as well as their own use of it.

*Because I got educated, if she has that education too and she understands about PrEP, I may allow her to use PrEP. Even if she would not inform me, I know about PrEP, if I found out that she is using it I would still allow her to use it*.

–Male partner, age 19, Dar Es Salaam

AGYW agreed that health providers would be well poised to deliver PrEP education in a convincing and non-judgmental manner to their partners.

*The only person who can speak to him freely is our doctor, even he has done something wrong… the best person to change his mind would be a doctor*.

–AGYW, age 24, Dar es Salaam

Both AGYW and male partners also noted that a broad-based education effort was needed to sensitize parents, peers, and the community at large about PrEP, all of whom were seen as key facilitators for PrEP uptake and adherence. AGYW noted that their family would be willing to support their PrEP use if they received adequate education enabling them to advocate for her use of it. Appropriate community sensitization efforts were also seen as the solution to combating widespread perceived HIV stigma.

*With education*, *the community will consider it [PrEP] as the normal medication…The other community can stigmatize him/her thinking that he/she has HIV… You know everybody tends to stick to what he knows already*.–AGYW, age 23, Mbeya

## Discussion

This study was among the first to highlight the perspectives of both AGYW and male partners of AGYW on the need for oral PrEP among heterosexual partners living without HIV, for HIV prevention program implementation and policy considerations. We used the framework for PrEP Introduction for AGYW [11] to guide the analysis of findings, focusing on partner dynamics and anticipated and perceived family and community stigma associated with PrEP use. AGYW saw PrEP as a particularly appealing HIV prevention option initiated and controlled independent of their partner, while male partners welcomed PrEP as a method of protecting themselves from admitted relationship infidelities. Both AGYW and male partners wanted to engage in dialogue with their partner to discuss and disclose their PrEP use, reduce distrust in their relationship, and proactively control their respective HIV risk. Anticipated HIV-related stigma associated with PrEP use was high among both AGYW and male partners, especially emanating from peers and family members as demonstrated by concerns about PrEP being mistaken for ARV medications and its implications towards promiscuity.

Desire for partner engagement and support in the decision-making to use PrEP is a key influencing factor related to Partner Dynamics in the PrEP framework [11] and operated within two dimensions. In the first dimension, which was an important consideration for all respondents, the desire and support emanated from each partner’s distrust of the other; that is, concerns about or knowledge of relationship infidelity. Even within the confines of a committed relationship, discussion and use of PrEP by partners is viewed as warranted due to relationship infidelities that increase both partners’ risk of HIV acquisition. A feasibility study among migrant miners in Mozambique and their female partners also found that most men wanted to be informed of their partner’s PrEP use due to mutual relationship distrust [36]. However, our study confirmed that despite admitted infidelities and high relationship distrust male partners are willing to support their AGYW partners to also use PrEP. In the second dimension, which was present for a few participants in both groups, the desire and support were means to reduce the negative effects from existing male partner control and unequal power within the relationship on the part of AGYW, while maintaining this control and power on the part of male partners. Several male partners, corroborated by a few AGYW, insisted that their partners must inform them of use of PrEP prior to initiation. Lack of informing or educating a male partner about PrEP could potentially result in emotional or physical violence as well as loss of financial resources. These are important, negative outcomes that carry serious physical and emotional well-being implications of PrEP disclosure. Pulerwitz and colleagues have also shown that gender norms inform power relationships which in turn influence the risks and opportunities for advancing SRH [19]. Vu et. al found that adolescents in Uganda often internalize gender norms about sexual and intimate relationships, underlying the need to confront unequal gender norms and related health outcomes [37].

Our study also demonstrates that for PrEP to be used effectively community sensitization efforts are needed to increase knowledge and decrease stigma around AGYW sexuality, a key determining factor of PrEP use from Mathur et al.’s framework [11]. Of importance in our study is the intersectional stigma that existed for AGYW and not for male partners. Both AGYW and male partners anticipated that their potential PrEP use would be associated with the stigma of being perceived as a person living with HIV, which is documented in clinical and demonstration trials among various populations [38, 39]. Our findings are similar to those in clinical trials where AGYW also noted stigma and lack of social support [40–42]. However, AGYW in our study also anticipated stigma linked to having sex; that is, they would be considered promiscuous if they use PrEP. Intersectional stigma is often discussed in the key population literature among MSM [43], sex workers [44] and PLHIV [45] but we saw a similar phenomenon emerge for AGYW. Intersecting stigmas can exacerbate the harmful effects of PrEP-related stigma, making those who are already disadvantaged less apt to seek HIV related health services [46]. Dismantling harmful social and intersecting stigmas will be crucial to supporting the uptake of HIV prevention methods by highly vulnerable populations.

### Recommendations for future research

These findings suggest that couples counseling, including adolescent couples, for PrEP uptake and adherence might be a well-placed strategy to educate partners about its shared benefits and potentially address unequal gender norms. PrEP counseling within sero-discordant couples on integrated PrEP and antiretroviral therapy delivery has been successful in encouraging mutual support and adherence to the medication as well as discussing sources of additional risk, such as new sexual partners, further stressing the importance of PrEP use and adherence [47, 48]. It is established in the HIV prevention literature that interventions focusing on both partners in the relationship, as opposed to just one partner, result in more consistent and higher uptake of the prevention methodology [49]. We posit that integrating couples PrEP counseling programs within couples HIV testing programs may help ensure buy-in and couples’ equal access to PrEP, stress the importance of its correct use and adherence, and reduce relationship distrust and other unhealthy relationship outcomes. The World Health Organization’s 2016 guidelines only make reference to PrEP adherence counseling [50] but not couples based counseling for sero-concordant couples. Similarly, informed choice counseling guidelines for women in Kenya and South Africa mention male partners only in the context of sero-discordant relationships, or women who are concerned that her partner has other sexual partners, as an indicator for when women should consider using PrEP [51]. Our findings are consistent with other studies that confirm gender and cultural norms often deny women the self-efficacy to access and use SRH products [52, 53], further emphasizing the need to deconstruct parallel gender norms and power dynamics among male partners of AGYW to better understand their support for PrEP.

As PrEP use becomes more widespread, community sensitization efforts should focus on dismantling harmful social and intersecting stigmas. PrEP marketing strategies need to consider the community, partner, and family contexts in which PrEP is introduced, as outlined in the PrEP introduction framework for AGYW [11]. Given the relative novelty of PrEP as it continues to be rolled out globally, socio-cultural dynamics and engagement of local communities is critical to effectively reaching AGYW and their male partners with PrEP [54, 55]. As more countries begin rolling out PrEP, tools such as the PrEP Communications Accelerator can be leveraged to create and market customized, high-quality strategic communication plans to help increase support and demand for PrEP among key target groups [56]. Additionally, outreach efforts should normalize PrEP use using messaging that allows users to feel proactive and informed about their choice of HIV prevention method to remove use associated stigma [57]. Approaches being used to reduce intersecting stigmas among PLHIV include developing multi-level resilience focused interventions that build social supports and coping mechanisms for women to mitigate the impacts of stigma at both the community and intrapersonal level [58–60]. Interventions from studies with PLHIV have also shown that providing gender sensitive training for health care workers, that allow them to reflect on their personal values and the impact of their negative attitudes, have increased client satisfaction and reduced reported stigma and discriminatory attitudes [61, 62]. Many of these approaches can be adapted to address anticipated stigma among PrEP users, particularly AGYW, and increase PrEP adherence.

### Limitations

Our exploratory study was conducted before PrEP became available in Tanzania. However, it does shed light on the complexities of relationship dynamics, specifically unequal power dynamics, and their associated health implications among AGYW and their male partners that should be explored further, especially amidst the current asynchronous targeting and rollout of PrEP among high-risk populations and its effect on PrEP uptake among other vulnerable populations, such as within the context of committed AGYW relationships. Subsequent PrEP research may want to explore how couple communication, distrust, gendered control, and attitudes toward HIV prevention influence PrEP use in various types of relationships, including casual and long-term committed partnerships among heterosexual couples who are living without HIV. This study did not explore differences in PrEP attitudes across various partnership categories, such as newer versus long-term partnerships or age disparate partnerships, however emerging literature from the region is showing the dire need to reduce HIV risk in transitional relationships [63]. Future work may benefit from examining when sexual partners enter into and transition out of committed relationships as possible PrEP entry points.

## Conclusions

In conclusion, increasing PrEP availability and acceptability in settings with a generalized epidemic should be prioritized as part of ongoing HIV prevention efforts. Combatting the HIV epidemic among vulnerable AGYW requires engaging both AGYW and male partners early on within the context of their relationships, particularly within couples counseling approaches, confronting unequal gender norms and their associated health implications, and promoting broad community awareness about PrEP among parents and peers to reduce stigma, including intersectional stigma, and support its mutual use. Findings from this exploratory study should be used to inform PrEP roll out to AGYW and their male partners in Tanzania and settings with similar gender and socio-cultural contexts.
